# Nickel oxide nanoparticles exposure as a risk factor for male infertility: “*In vitro*” effects on porcine pre-pubertal Sertoli cells

**DOI:** 10.3389/fendo.2023.1063916

**Published:** 2023-03-30

**Authors:** Iva Arato, Stefano Giovagnoli, Alessandro Di Michele, Catia Bellucci, Cinzia Lilli, Maria Chiara Aglietti, Desirée Bartolini, Angela Gambelunghe, Giacomo Muzi, Mario Calvitti, Elena Eugeni, Francesco Gaggia, Tiziano Baroni, Francesca Mancuso, Giovanni Luca

**Affiliations:** ^1^ Department of Medicine and Surgery, University of Perugia, Perugia, Italy; ^2^ Department of Pharmaceutical Sciences, University of Perugia, Perugia, Italy; ^3^ Department of Physics and Geology, University of Perugia, Perugia, Italy; ^4^ Internal Medicine Endocrine and Metabolic Sciences Unit, Santa Maria della Misericordia Hospital of Perugia, Perugia, Italy; ^5^ International Biotechnological Center for Endocrine, Metabolic and Embryo-Reproductive Translational Research (CIRTEMER), Department of Medicine and Surgery, University of Perugia, Perugia, Italy; ^6^ Division of Medical Andrology and Endocrinology of Reproduction, Saint Mary Hospital, Terni, Italy

**Keywords:** Sertoli cells, nickel oxide nanoparticles, ROS, comet, MAPK pathways

## Abstract

Lately, nickel oxide nanoparticles (NiO NPs) have been employed in different industrial and biomedical fields. Several studies have reported that NiO NPs may affect the development of reproductive organs inducing oxidative stress and, resulting in male infertility. We investigated the *in vitro* effects of NiO NPs on porcine pre-pubertal Sertoli cells (SCs) which undergone acute (24 h) and chronic (from 1 up to 3 weeks) exposure at two subtoxic doses of NiO NPs of 1 μg/ml and 5 μg/ml. After NiO NPs exposure we performed the following analysis: (a) SCs morphological analysis (Light Microscopy); (b) ROS production and oxidative DNA damage, gene expression of antioxidant enzymes (c) SCs functionality (AMH, inhibin B Real-time PCR analysis and ELISA test); (d) apoptosis (WB analysis); (e) pro-inflammatory cytokines (Real-time PCR analysis), and (f) MAPK kinase signaling pathway (WB analysis). We found that the SCs exposed to both subtoxic doses of NiO NPs didn’t sustain substantial morphological changes. NiO NPs exposure, at each concentration, reported a marked increase of intracellular ROS at the third week of treatment and DNA damage at all exposure times. We demonstrated, un up-regulation of SOD and HO-1 gene expression, at both concentrations tested. The both subtoxic doses of NiO NPs detected a down-regulation of AMH and inhibin B gene expression and secreted proteins. Only the 5 μg/ml dose induced the activation of caspase-3 at the third week. At the two subtoxic doses of NiO NPs a clear pro-inflammatory response was resulted in an up-regulation of TNF-α and IL-6 in terms of mRNA. Finally, an increased phosphorylation ratio of p-ERK1/2, p-38 and p-AKT was observed up to the third week, at both concentrations. Our results show the negative impact of subtoxic doses NiO NPs chronic exposure on porcine SCs functionality and viability.

## Introduction

Recent advancements in the discipline of nanotechnology have introduced the employment of engineered nanoparticles (NPs) in the production systems of numerous consumer products, as well as in various industrial applications and some innovative medical practices.

NPs are also widespread in the environment and they can proceed to enter the human body *via* inhalation, ingestion, skin adsorption, and *via* intravenous injection when used for medical applications (1).

One of the most common metal nanomaterial, nickel oxide nanoparticles (NiO NPs) have found broad application prospects in many fields, such as magnetism, energy technology and biomedicine and have therefore attracted great interest. In the biomedical field, NiO NPs can be used in many ways, like creation of biological probes, isolation of DNA from total proteins, targeted drug delivery, treatment of malignant tumor cells with hyperheat, contrast-enhanced magnetic resonance imaging (2).

In the last decade, various studies have reported on the dangers of exposure to NiO NPs that have been found to induce pulmonary toxicity (3–5), liver and spleen toxicity (6, 7) cardiovascular toxicity (8), genotoxicity (9, 10) and spermiotoxicity (11) and, even to induce cancer (6, 7, 9, 12).

Notably, the lung toxicity of NiO NPs was greatly investigated, demonstrating relevant inflammatory, cytotoxicity and apoptotic effects on the alveolar cells (13, 14). Other studies highlight that NiO NPs can reach the gastrointestinal tract through the unintended ingestion of food and water contaminated with NiO (15).

Studies by Saquib et al. estabilished that NiO NPs may interest many different tissues of male wistar rats, resulting in genotoxicity and imbalanced enzymes activity (16). The NiO NPs were described as an hepatotoxic agent due to their ability to induce oxidative stress and apoptosis in the human liver cells (17).

Singh et al. reported that NiO NPs may also induce a state of oxidative imbalance in the testes of male rats after oral exposure, leading to DNA damage and subsequent infertility (18).

Recently, assessing male reproductive health and infertility, one of the most crucial problem caused by environmental pollution, has attracted increased attention from many scientists.

It has been hypothesized that exposure to NiO NPs through industrial use or occupational environment may compromise the reproductive system; in fact several studies reported that NiO NPs has reproductive and developmental toxicity (11, 19–23). In an adult albino rat model, it has been estabilished that nanomaterials can easily cross the blood-testis barrier and can cause germ cell damage due to their properties related to their size (23). To date, however, the precise mechanism that correlates Nickel NPs exposure with testicular damage is not entirely clear, still it is relevant that mitochondrial damage has been shown to be an important player in altered sperm parameters and damage to Leydig cells and testis (24).

In the present study, we focused our attention on the *in vitro* effects of NiO NPs on primary cultures of Sertoli cells (SC).

SC are a key element in spermatogenesis through their ability to support stem cells, providing both nourishment and physical support. They are also able to protect them from the host immune system, either through the formation of an SC- based blood- testis barrier (BTB) or through the release of numerous immunomodulatory factors.

Their main contribution to the unfolding of spermatogenesis is characterized by the production of critical factors necessary for the successful development of spermatogonia, throughout the stage of mature spermatozoa (25, 26).

Our experimental studies conducted in the last years have enabled us to develop a system, using *in vitro* pre-pubertal porcine bioengineered cell culture system as a new model for experimental studies on male infertility. We have successfully isolated pure and functional porcine pre-pubertal SC preparations (SCs), preferred to adult cells since the latter are very difficult, if not impossible, to isolate.

The ability to obtain functional SCs was demonstrated by their secretion of Anti-Mullerian hormone (AMH) and inhibin B, as key functional parameters of superior mammalian SCs, after follicle stimulating hormone (FSH) stimulation (27).

Our primary SCs cultures show many similarities to human SC and hold great potential as an experimental model to study the “*in vitro*” effects of toxic substances and heavy metals, as demonstrated in our previous work that confirmed the negative impact of Titanium dioxide NPs on SCs, and our works on toxicity of cadmium and lead (28–30).

The goal of the present study was to evaluate the influence of acute (24 h) and chronic (from 1 up to 3 weeks) exposure to subtoxic doses NiO NPs of 1 μg/ml and 5 μg/ml on our “*in vitro*” model of SCs.

## Materials and methods

### Preparation and characterization of NiO NPs

Chemicals: Ni(CH_3_COO)_2_·_2_H_2_O, and NH_4_HCO_3_ were purchased from Sigma-Aldrich.

For the synthesis of NiO NPs 50 mL of a NH_4_HCO_3_ 1 M solution were dropped, under ultrasound irradiation for 15 minutes at 50% of amplitude at 25°C, to a 100 mL of a Ni(CH_3_COO)_2_·_2_H_2_O 4x10^-3^ M solution. The pH was maintained at 9. The high power ultrasound irradiation was carried out by an Ultrasonic processors VC750 Sonics and Materials, 20 kHz with a diameter tip of 13 mm. The precipitate was dried, calcined at 350°C for 1 h and characterized by ICP-OES, FE-SEM-EDX and XRD (31).

### SCs culture and NiO NPs exposure

Animal studies were conducted in agreement with the guidelines adopted by the Italian Approved Animal Welfare Assurance (A-3143-01) and the European Communities Council Directive of November 24, 1986 (86/609/EEC). The experimental protocols were approved by the University of Perugia. Number 3 Danish Duroc pre-pubertal pigs (15 to 20 days old) underwent bilateral orchidectomy after general anaesthesia with ketamine (Ketavet 100; Intervet, Milan, Italy), at a dose of 40 mg/kg, and dexmedetomidine (Dexdomitor, Orion Corporation, Finland), at a dose of 40 g/kg, and were used as SCs donors. Specifically, pure porcine pre-pubertal SCs were isolated, and characterized according to previously established methods (27).

In detail, SCs have undergone acute (24 h) and chronic (from 1 up to 3 weeks) exposures at two subtoxic doses of NiO NPs of 1 μg/ml and 5 μg/ml according to 3-(4, 5-Dimethyl-thiazol-2-yl)22,5-diphenyl-tetrazolium bromide (MTT) assay. The control group consisted of unexposed SCs (0 NiO NPs μg/ml).

### ICP-OES

NiO NPs -treated SCs were detached by trypsin/ethylenediaminetetraacetic acid (EDTA) (Lonza, Verviers, Belgium) at 37°C for 8 min, to promote the enzymatic reaction. After washing with 1 ml Hank’s balanced salt solution (HBSS) (Sigma-Aldrich Co., St. Louis, MO, USA), samples were centrifuged at 150×g for 6 min, the supernatant was removed, the pellets were freeze-dried, and accurately weighed. Samples were dissolved by treatment with 10 ml of a mixture of sulfuric acid (H_2_SO_4_), (97% Sigma-Aldrich Co., St. Louis, MO, USA)/nitric acid (HNO_3_ 70%), (Sigma-Aldrich Co.,St. Louis, MO, USA) (2:1). After solubilization, the obtained solutions were diluted with the EDTA solution (1:10) prior Ni2+ content determination using a Varian 700-Es series spectrometer (Agilent, Milan, Italy) in triplicate. Calibration was performed diluting a Nickel nitric acid stock solution for ICP (Sigma Aldrich, Milan, Italy) to obtain Nickel standard solutions in the 1–15-mg/ml range. The Ni2+ uptake in SCs was calculated per unit weight of freeze-dried NiO NP-treated SCs and % of the total amount added and the error expressed as SEM.

### MTT assay and cell viability

NiO NPs cytotoxicity was evaluated by the MTT (Sigma-Aldrich Co., St. Louis, MO, USA) test on unexposed and exposed SCs. Briefly NiO NPs at the concentrations of 2.5, 5, 15, 30, 45, 60 and 120 μg/ml were added to each well and cultured for additional 24 or 48 h. Then, the experiment was performed, as previously reported (28). Unexposed (0 NiO NPs μg/ml) SCs served as controls. Viability was expressed as a percentage with respect to unexposed SCs (NPs-exposed SCs ×100/unexposed SCs). The sub-toxic doses of 1 and 5 μg/ml were chosen for all subsequent experiments at 24 hours (acute exposure) and 1, 2, 3 weeks (chronic exposure) and MTT assay was performed at each experimental time-point.

### ROS determination

Intracellular ROS were measured by treating unexposed and exposed SCs with 50 mM dichlorofluorescein diacetate (DCFHDA) (Sigma-Aldrich Co., St. Louis, MO, USA) solution in Dulbecco’s phosphate-buffered saline (D-PBS) (Sigma-Aldrich Co., St. Louis, MO, USA) at 37°C for 30 min. Fluorescence was read by using a plate reader (DTX 880 Multimode Detector, Beckman Coulter). Data were normalized for cell viability (MTT assay) and expressed as the percentage of unexposed SCs. The sensitivity of the test was confirmed by adding 30 μM hydrogen peroxide (H2O2) (30 min) on unexposed SCs as positive control.

### Oxidative DNA damage quantification

To evaluate the oxidative DNA damage, unexposed and exposed SCs were processed in the comet assay under alkaline conditions (alkaline unwinding/alkaline electrophoresis, pH >13), basically following the original procedure (32). Briefly, SCs treated with 1 mM 4-nitroquinoline N-oxide (4NQO) (Sigma-Aldrich, Milan, Italy) for 1 h at 37°C (33) were used as positive control. At the end of treatments, the cells were detached with trypsin (Invitrogen, Milan, Italy) and collected by centrifugation (70×g, 8 min, 4°C). Then, cell pellets were gently resuspended in low-melting point agarose (Sigma-Aldrich, St. Louis, MO, USA) at 37°C, layered onto a conventional microscope slide precoated with 1% normal melting point agarose and covered with a coverslip (Knittel-Glaser, Braunschweig, Germany). Then, electrophoresis runs were then performed as previously reported (28).

The comets in each microgel were analysed (blind), at ×500 magnification with an epi-fluorescent microscope (BX41, Olympus, Tokyo, Japan), equipped with a high sensitivity black and white charge-coupled device (CCD) camera (PE2020, Pulnix, UK), under a 100-W high-pressure mercury lamp (HSH-1030-L, Ushio, Japan), using appropriate optical filters (excitation filter 510–550 nm and emission filter 590 nm). Images were elaborated by Comet Assay III software (Perceptive Instruments, UK). A total of 100 randomly selected comets (50 cells/replicate slides) were evaluated for each experimental point.

### AMH and inhibin B secretion assays

Aliquots of culture media from all the experimental groups were collected and stored at -20°C for subsequent assessment of AMH (AMH Gen II ELISA, Beckman Coulter, Webster, TX, USA) and inhibin B (inhibin B Gen II ELISA, Beckman Coulter) secretion levels as previously described (34).

### Reverse transcriptase‐polymerase chain reaction analysis

AMH, inhibin B, TNF-α,IL-6, SOD1, HO-1, GHSPx and NRF2 were analysis by reverse transcriptase‐polymerase chain reaction (RT‐PCR) as previously described in Arato et al. (35) employing the primers listed in **Table 1**.Total RNA was extracted using the TRIzol reagent (Sigma‐Aldrich), and quantified by reading the optical density at 260 nm. In detail, 2.5 μg of total RNA was subjected to reverse transcription (RT, Thermo Scientific) to a final volume of 20 μl. We performed the qPCR with the use of 25 ng of the cDNA obtained by RT and an SYBR Green Master Mix (Stratagene). This procedure was performed in an Mx3000P cycler (Stratagene), using FAM for detection and ROX as the reference dye. We normalized the mRNA level of each sample against β‐actin mRNA and expressed it as fold changes versus the levels in the control group.

**Table 1:** Primer sequences for PCR analyses.

Gene	Forward sequences (5′–3′)	Reverse sequences (5′–3′)
AMH	GCGAACTTAGCGTGGACCTG	CTTGGCAGTTGTTGGCTTGATATG
Inhibin B	CCGTGTGGAAGGATGAGG	TGGCTGGAGTGACTGGAT
SOD1	TCGGGAGACCATTCCATCAT	ACCTCTGCCCAAGTCATCT
HO-1	CTGGTGATGGCGTCCTTGTA	TTGTTGTGCTCAATCTCCTCCT
GHSPx	CGAGAAGTGTGAGGTGAATGG	GCGGAGGAAGGCGAAGAG
NRF2	TTCACTAAACCCAAGTCCCAGCAT	AAGCCAAGCAGTGTGTCTCCATA
IL-6	AATGCTCTTCACCTCTCC	TCACACTTCTCATACTTCTCA
TNF-α	CTCTTCTCCTTCCTCCTG	GCTTTGACATTGGCTACA
β-actin	ATGGTGGGTATGGGTCAGAA	CTTCTCCATGTCGTCCCAGT

AMH, anti-Müllerian hormone; SOD1, superoxide dismutase 1; HO-1, heme-oxygenase 1; GHSPx, gluthatione peroxidase; NRF2, Nuclear factor erythroid 2-related factor 2; IL-6, interleukin-6; TNF-α, Tumor necrosis factor-alpha.

### Immunoblot

Total protein extracts were prepared for immunoblot analysis as described in Mancuso et al. (36). Briefly, the cell extracts were separated by 4–12% SDS-PAGE, and then blotted on nitrocellulose membranes (BioRad, Hercules, CA, USA). The membranes were incubated overnight in a buffer containing 10 mM TRIS (Sigma-Aldrich Co., St. Louis, MO, USA), 0.5 M NaCl (Sigma-Aldrich Co., St. Louis, MO, USA), 1% (v/v) Tween 20 (Sigma-Aldrich Co., St. Louis, MO, USA), rabbit anti-ERK1/2 (Millipore, MA, USA, 1:2000), mouse anti-phospho- ERK1/2 (Millipore; MA, USA, 1:100), rabbit anti-JNK (Millipore; MA, USA, 1:1000), rabbit anti-phospho-JNK (Millipore; MA, USA, 1:500), rabbit anti-posphop38 (Millipore, MA, USA, 1:2000), mouse anti p38 (Millipore, MA, USA, 1:2000), anti-Akt (Cell Signalling, 1:100), rabbit anti-phospho-Akt (Cell Signalling, 1:1000), rabbit anti-phospho-NF-kB p65 antibody (AbCam, Cambridge, UK, 1:1000), rabbit anti-NF-kB p65 antibody (AbCam, Cambridge, UK, 1:1000), and mouse anti-β-actin (Sigma-Aldrich Co., St. Louis, MO, USA, 1:100) primary antibodies.

Primary antibody binding was then detected by incubating membranes for an additional 60 min in a buffer containing horseradish peroxidase conjugated anti-rabbit (Sigma-Aldrich Co., St. Louis, MO, USA,1:5000) and/or anti-mouse (Santa Cruz Biotechnology Inc., 1:5000) IgG secondary antibodies. The bands were detected by enhanced chemiluminescence and acquired by ChemiDoc imaging System (Bio-Rad, Hercules, CA, USA).

### Data analysis

Normality analysis was performed by Shapiro–Wilk test, and statistical comparisons were analyzed using one-way ANOVA followed by Tukey’s HSD *post hoc* test (SigmaStat 4.0 software,Systat Software Inc., CA, USA). Values were reported as the means ± SEM of three independent experiments, each performed in triplicate. Differences were considered statistically significant at *p < 0.05, and **p < 0.001 compared to unexposed SCs (0 NiO NPs).

## Results

### Characterization of NiO NPs

**Figure 1:**
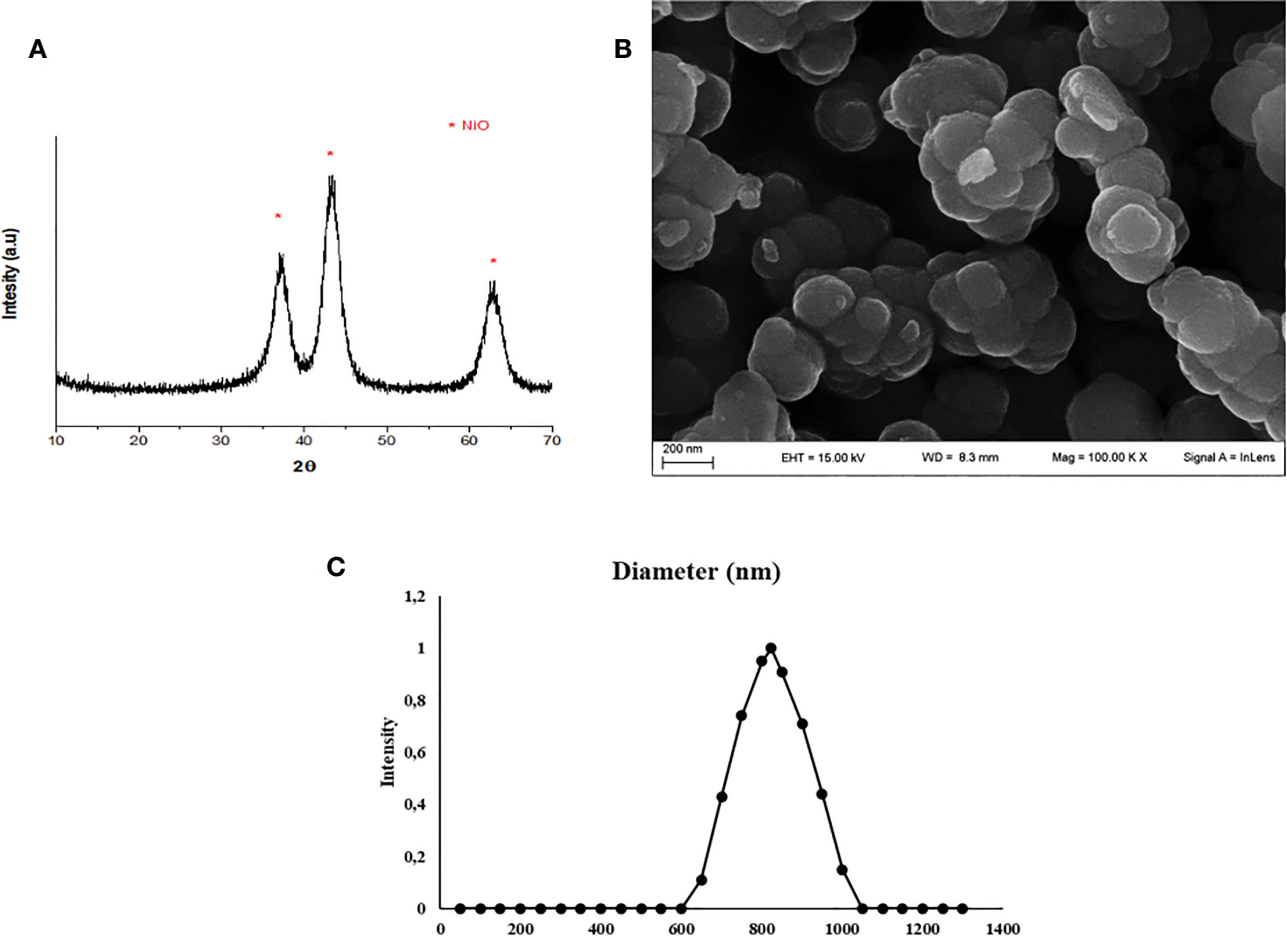
NiO NPs characterization. **(A)** XRD analysis: diffractogram, * represents the peaks of NiO NPs in anatase form (JCPDS 00-001-0562). **(B)** SEM analysis: the mean size distribution of TiO2 NPs in dry form was 20 ± 5 nm. **(C)** DLS analysis: mean hydrodynamic diameter of TiO2 NPs at 10 μg/ml in culture medium.

The synthesis of NiO NPs was performed by XRD analysis, the resulting diffractogram was characteristic of nichel oxide crystals in anatase form (JCPDS 00-001-0562) (**Figure 1A**).

A representative SEM image of NiO NPs in dry form is showed in **Figure 1B**, and the mean size distribution reports values of 20 ± 5 nm diameter, calculated by measuring over 100 particles in random fields of view. Results showed that NiO NPs tended to form aggregates of submicrometric dimensions. DLS analysis, confirmed some aggregation of NiO NPs in suspension. The mean hydrodynamic diameter of NiO NPs mainly distributed in a range of 100–800 nm (**Figure 1C**).

### Uptake of NiO NPs by SCs

ICP-OES was used to quantify the uptake of NPs expressed as the percentage of internalized NPs and the amount of metal adsorbed per cell number (expressed as ng/10^5^), at each concentration, after 5 hours of treatment (**Figure 1S**). In the treatment after 5 hours, the percentage of internalized NPs showed a range between 1 and 3% (**Figure 1S**), where the lower treatment dosage (1µg/ml) exhibited a higher percentage of uptake than the higher dosage (5µg/ml). On the contrary, the amount absorbed and expressed as ng/10^5^ was much higher at the higher dosages (1.57 for NiO).

We could speculate that this difference between percentage and net amount absorbed was probably due to the gradual saturation of SCs, leading to a progressive slowing down in uptake as the concentration of NPs increases, further confirming literature data (37).

Other factors that could have negatively influenced the absorption of NiO particles are their marked hydrophobicity and their tendency to stick to surfaces, phenomena due to the high surface energy of these particles that causes a reduced availability for absorption by part of the SCs (38).

### NiO NPs cytotoxicity evaluation

**Figure 2:**
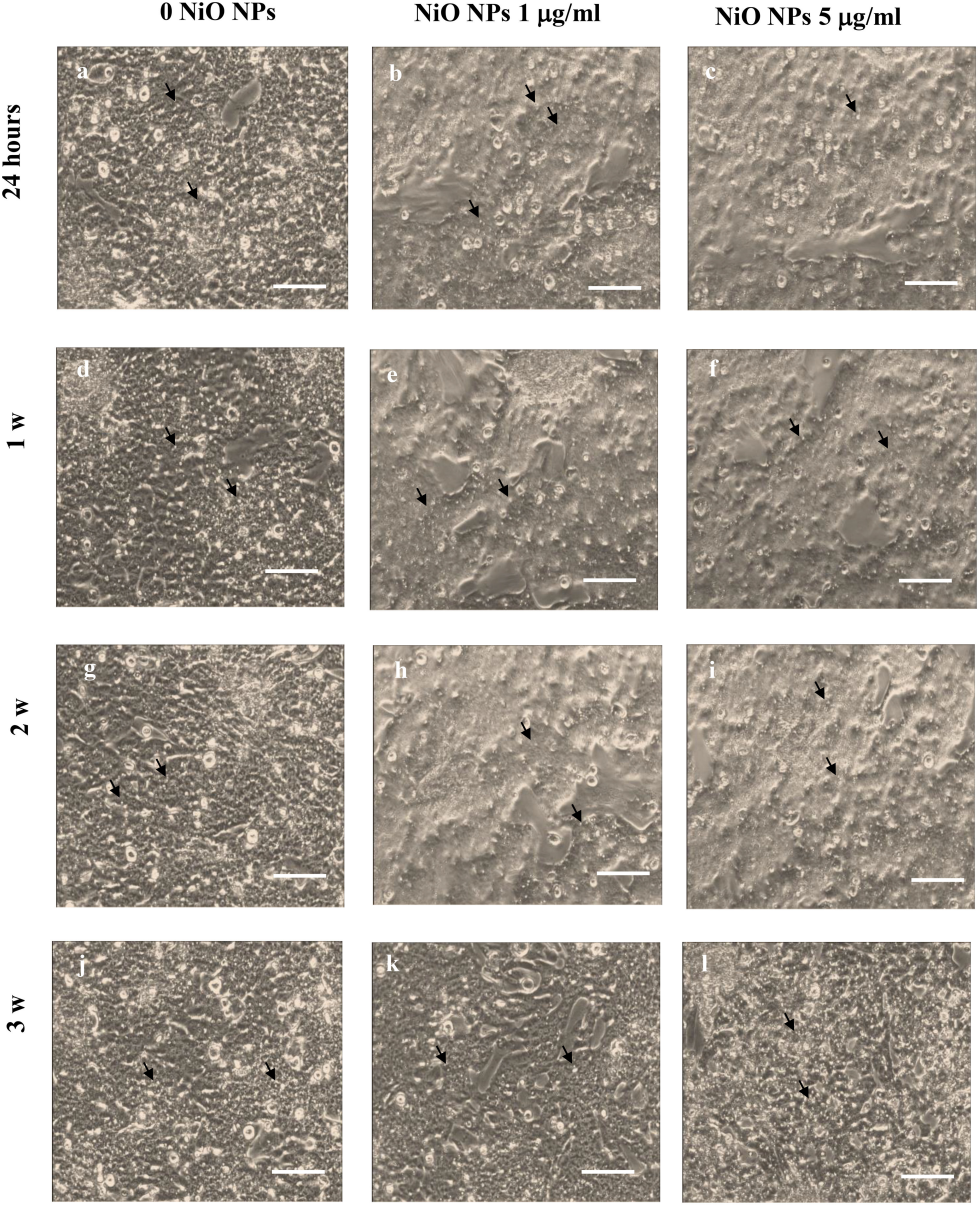
SCs Morphological characterization. Light microscope of unexposed (0 NiO NPs) SCs **(A, D, G, J)**, NiO NPs exposed SCs at 1 **(B, E, H, K)**, and 5 μg/ml **(C, F, I, L)** at 24 h **(A–C)** and 1 **(D–F)**, 2 **(G–I)**, and 3 weeks **(J–L)**. Black arrows point to some of the abundant vacuoles containing lipid hormones, likely testosterone coniugated with androgen binding protein (ABP) and estradiol. The scale bar corresponds to 200 μm for **(A–L)**. The images are representative of three separate experiments.

For the preliminary study, at 24 hours, as shown in **Figure 2S**, panel A, NiO NPs at low concentrations appeared to induce proliferation of SCs, probably due to an adaptive response to the increasing NPs concentration. Proliferative effects due to metal NPs have been found many times before and this has been attributed to the activation of specific pathways, such as MAP kinases (28), but many aspects remain to be elucidated. The percentage of metabolically active cells began to decline at doses of 40 μg/ml (*p< 0.05 vs. unexposed SCs), showing a LD50 of 80 μg/ml (**p<0.001 vs. unexposed SCs). Finally, a collapse in SCs viability was also identified at 100 and 120 μg/ml (**p<0.001 vs. unexposed SCs). At 48 hours, a statistically significant reduction in metabolic activity was identified, in SCs exposed to a concentration of 40 μg/ml compared to controls (*p< 0.05 vs. unexposed SCs), with a LD50 dropping to 70 μg/ml (**p<0.001 vs. unexposed SCs), followed by a drastic reduction in viability at higher concentrations (**Figure 2S**). Due to the obvious toxicity of NiO NPs, for the 3-week treatment the sub-toxic concentrations of 1 μg/ml and 5 μg/ml, were chosen, the former to simulate a mild exposure to seemingly harmless concentrations, to which humans could easily be exposed in everyday life, while the latter represents a higher dosage to allow an evaluation of possible mechanisms of toxicity.

MTT assays performed during the 3 weeks of treatment with NiO NPs showed an increase in metabolically active cells at the dosage of 5 μg/ml at 1 week (**Figure 2S** panel B *p<0.05 vs. unexposed SCs). This effect could be due to a defensive mechanism put in place by the cell in response to a noxious stimulus, however it does not seem to be sufficient to preserve the cell from the statistically significant reduction in the percentage of metabolically active cells of 10% observed at the third week compared to the unexposed SCs (**Figure 2S** panel B, *p<0.05 vs. unexposed SCs).

In contrast, at the concentration of 1 μg/ml the only significant effect was observed at the second week with a 10% loss of viability (*p<0.05 vs. unexposed SCs) which then recovered at the third week, demonstrating the low level of toxicity potential at this concentration, which allowed the cell to recover from the stress suffered (**Figure 2S** panel B).

### SCs light microscopy

Morphological analysis revealed that SCs exposed to both subtoxic doses of NiO NPs did not undergo substantial changes compared with the untreated monolayer at all times of exposure, shown in the **Figures 2A, D, G, J**. In fact, cells exposed to NiO NPs maintained the typical squamous shape of epithelial cells with vacuoles containing lipid hormones, likely testosterone coniugated with androgen binding protein (ABP) and estradiol, well evident and abundantly distributed in the cell surface (**Figures 2B, C, E, F, H, I, K, L**).

### Impact of NiO NPs on the liveness of ROS and oxidative DNA damage

**Figure 3:**
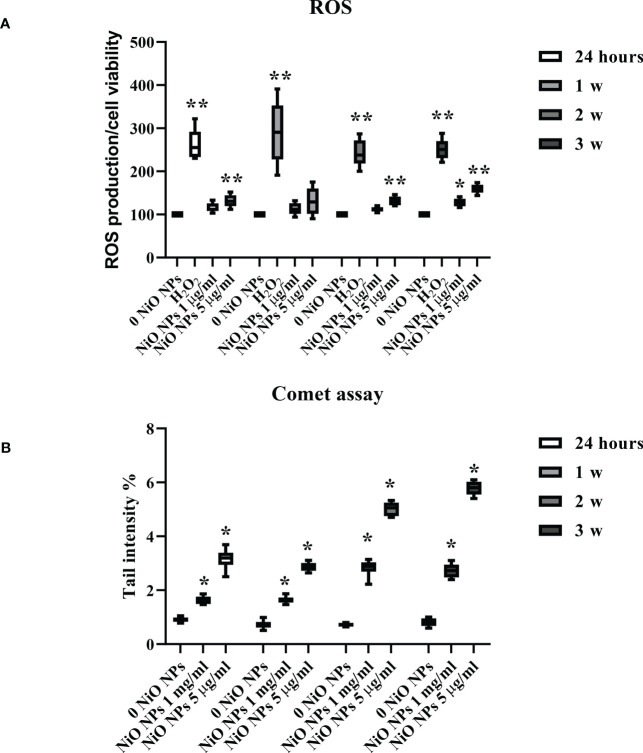
ROS production and DNA damage after NiO NPs treatment in SCs. **(A)** Total intracellular ROS production in SCs exposed to NiO NPs 1 and 5 μg/ml for 24 h and 1, 2, and 3 weeks. Data represent the mean ± SEM (**p < 0.001 with respect to 0 NiO NPs of three independent experiments). **(B)** DNA damage expressed as tail intensity % in unexposed SCs and exposed to NiO NPs and 5 μg/ml for 24 h and 1, 2, and 3 weeks. Data represent the mean ± SEM (*p < 0.05 vs. 0 NiO NPs of three independent experiments).

As shown in **Figure 3A**, the dose of 1 μg/ml NiO NPs did not affect ROS intracellular level up to 2 weeks post exposure. On the contrary, at 3 weeks post treatment, ROS level significantly increased compared to unexposed SCs. Conversely, the dose of 5 μg/ml NiO NPs induced a significant increase of intracellular ROS amounts over time and until the end of treatment compared to unexposed SCs (**Figure 3A**, **p < 0.05 **p < 0.001 vs. 0 NiO NPs). As expected, H_2_O_2_ (positive control) induced a significant increase in ROS intracellular levels (**Figure 3A**, **p < 0.05 **p < 0.001).

The levels of oxidative DNA damage induced by NiO NPs were measured as the % of DNA in tail by the alkaline comet assay over time, after acute (24 h) and chronic exposures (from 1 to 3 weeks) to 1 and 5 μg/ml of NiO NPs. Both doses of exposure, induced a significant increase in the oxidative DNA damage over time and until the end of treatment with respect to the unexposed SCs (**Figure 3B**, **p < 0.05 vs. 0 NiO NPs).

### Antioxidant response

**Figure 4:**
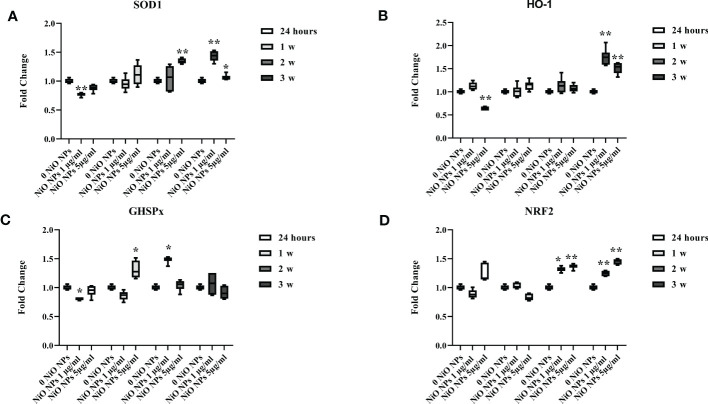
Real-time PCR analysis of antioxidant and metabolic enzymes. Gene expression of SOD1 **(A)**, HO-1 **(B)**, GHSPx **(C)**, and NRF2 **(D)** in SCs at 24 h and 1, 2, and 3 weeks of incubation with NiO NPs 1 and 5 μg/ml. Data represent the mean ± SEM (*p < 0.05, **p < 0.001 vs. 0 NiO NPs of three independent experiments, each performed in triplicate).

The gene expression of SOD1 significant increased at the dose of 1 μg/ml only at the third week of treatment, meanwhile at the dose of 5 μg/ml it showed a significant increase from second up to third week (**Figure 4A**, *p < 0.05 and **p < 0.001 vs. 0 NiO NPs).

The gene expression of HO-1 increased at both concentrations only at the third week of NiO NPs-exposure (**Figure 4B**, **p < 0.001 vs. 0 NiO NPs).

We observed a significant increase in GHSPx gene expression only at the second week at the dose of 1 μg/ml and, at first week at the dose of 5 μg/ml; with a significant reduction at 24h at the dose of 1 μg/ml (**Figure 4C**, *p < 0.05 vs. 0 NiO NPs).

The gene expression of NRF2 showed a significant increase at both dose from second up to third week of treatment (**Figure 4D**, *p < 0.05 and **p < 0.001 vs. 0 NiO NPs).

### NiO NPs effects on SCs functionality

**Figure 5:**
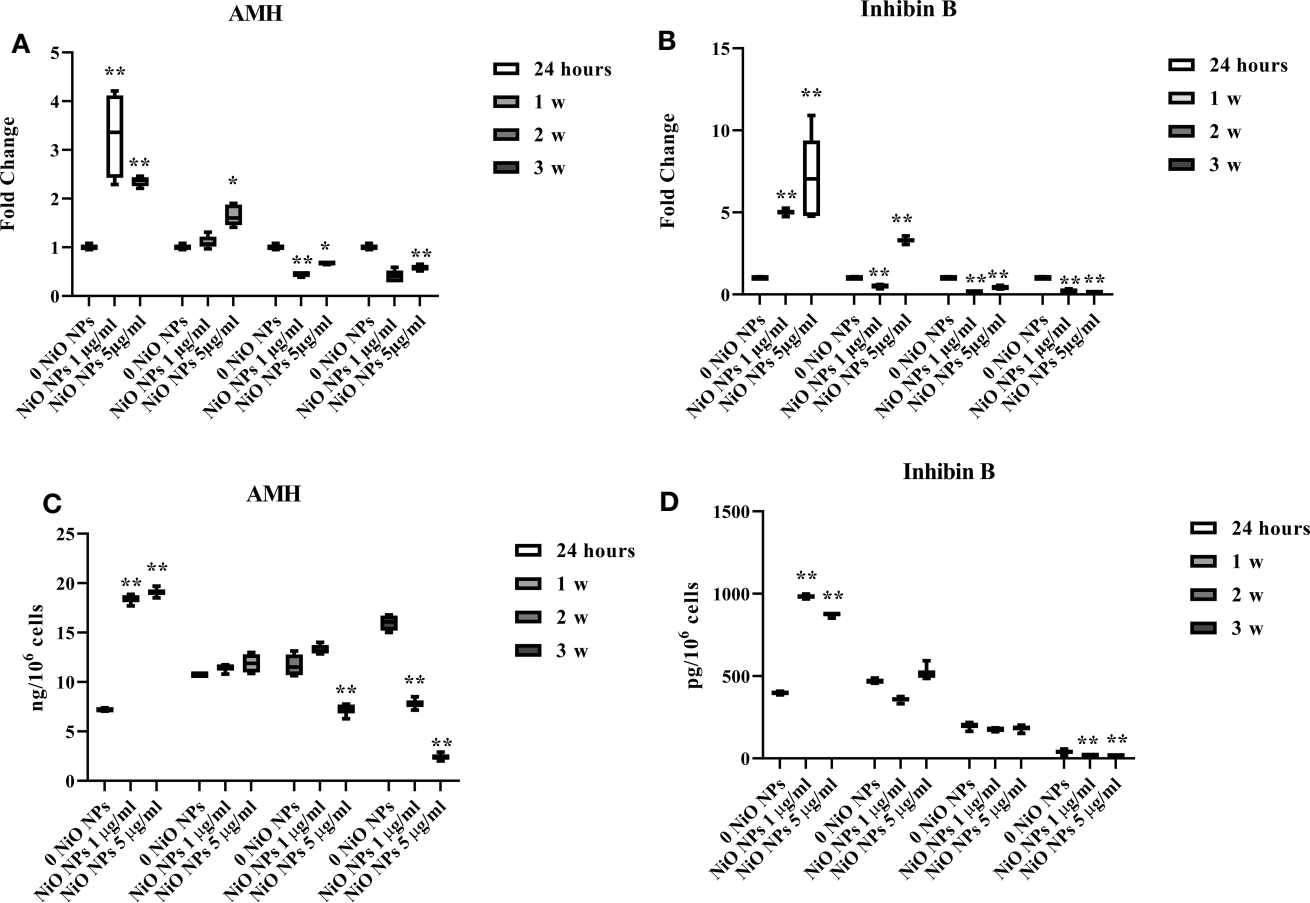
Effect of NiO NPs on SCs functionality parameters AMH and inhibin Gene expression of AMH **(A)**, and inhibin B **(B)** in SCs at 24 h and 1, 2, and 3 weeks of incubation with NiO NPs 1 and 5 μg/ml. Data represent the mean ± SEM (*p < 0.05 and **p < 0.001 vs. 0 NiO NPs of three independent experiments, each performed in triplicate). ELISA assay of **(C)** AMH and **(D)** inhibin B secretion in SCs at 24 h and 1, 2, and 3 weeks of incubation with NiO NPs 1 and 5 μg/ml. Data represent the mean ± SEM (**p < 0.001 vs. 0 NiO NPs of three independent experiments, each performed in triplicate).

Exposure of SCs to both concentrations of NiO NPs induced a significant increase in AMH and inhibin B gene expression at 24 h, followed by a significant decrease after 2 week up to the third week, compared to unexposed SCs (**Figures 5A, B** *p < 0.05 and **p < 0.001 vs. 0 NiO NPs).

At both concentrations, AMH and inhibin B secretion was significantly increased at 24h of treatment. The secretion of AMH was significantly decreased after dose of 1 μg/ml NiO NPs only the third week, whereas at 5 μg/ml NiO NPs, we observed a significant reduction from the second, up to the third week of treatment respect to unexposed SCs (**Figure 5C** **p < 0.001 vs. 0 NiO NPs).

In contrast, inhibin B secretion was significantly decreased after exposure to both dose of treatment only at third week with respect to unexposed SCs (**Figure 5D** **p < 0.001 vs. 0 NiO NPs).

### Caspase-3 evaluation

We observed that NiO NPs exposure, at each concentration, induced the activation of caspase-3 at third week, with the cleavage of p19 and p17 fragments p19 kDa active fragment.

**Figure 6:**
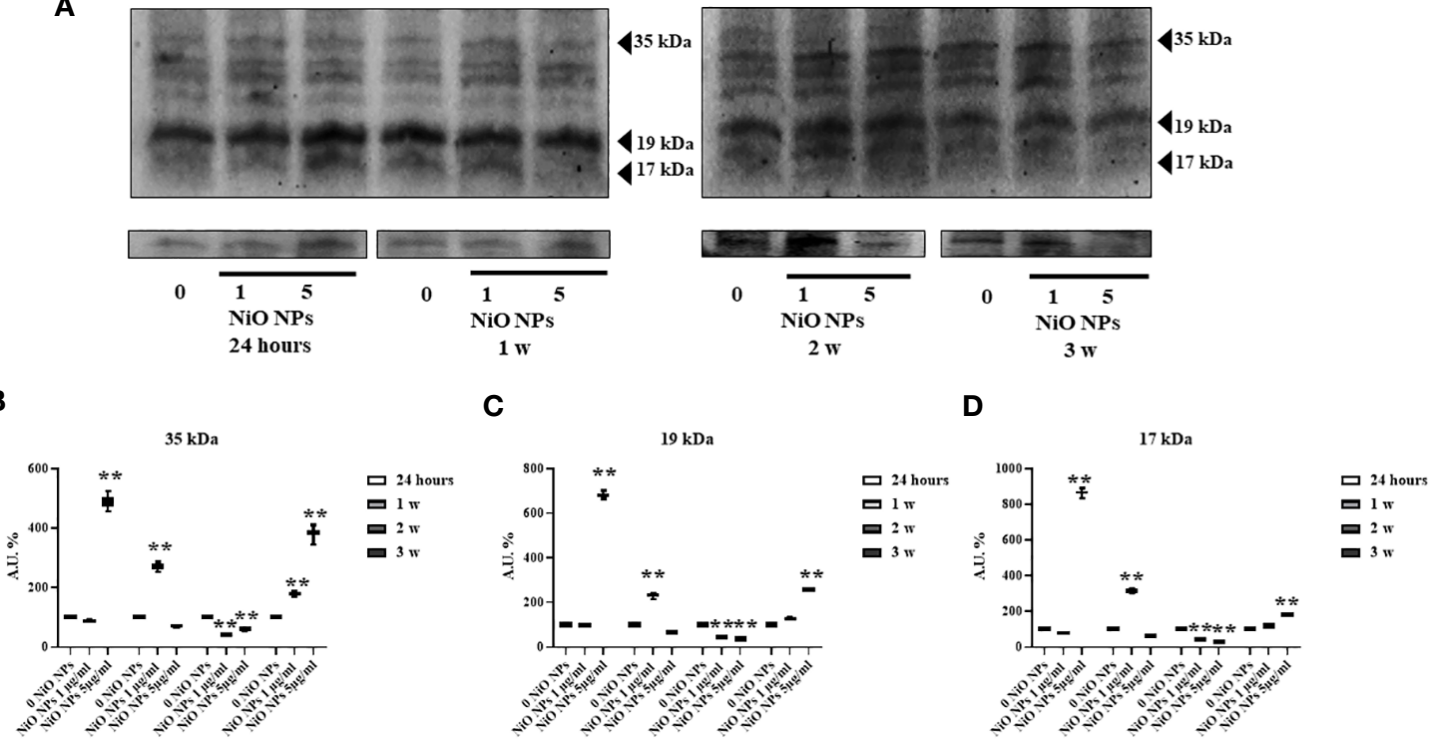
Caspase-3 Evaluation by WB analysis. **(A)** Immunoblots of caspase-3 p35, p19, and p17 in SCs at 24h and 1, 2, and 3 weeks of incubation with NiO-NPs at 1 and 5mg/ml. Densitometric analysis of the protein bands of caspase-3 p35 **(B)**, p19 **(C)**, and p17 **(D)** in SCs at 24 h and 1, 2, and 3 weeks of incubation with NiO NPs 1 and 5 μg/ml. Data represent the mean ± SEM (**p < 0.001 vs. 0 NiO NPs of three independent experiments, each performed in triplicate).

Only at the dose of 5 μg/ml NiO NPs, we demonstrated a statistical increase of both active p19 and p17 with respect to the inactive p35 fragments, expression of a more prominent apoptotic process (**Figures 6A–D** **p < 0.001 vs. 0 NiO NPs).

### Pro-inflammatory response

**Figure 7:**
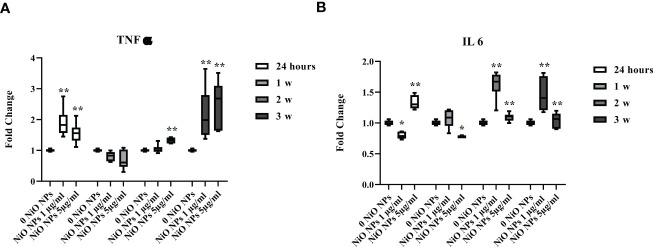
Real-time PCR analysis of SCs proinflammatory response. Gene expression of TNF-α **(A)**, IL-6 **(B)** in SCs at 24 h and 1, 2, and 3 weeks of incubation with NiO NPs 1 and 5 μg/ml. Data represent the mean ± SEM (*p < 0.05, **p < 0.001 vs. 0 NiO NPs of three independent experiments, each performed in triplicate).

At both concentration, the gene expression of TNF-α showed a significant increase at 24 h and during third week, (**Figures 7A**, **p < 0.001 vs. 0 NiO NPs).

Moreover, IL-6 gene expression showed a significant increase at 24 h only at dose of 5 μg/ml NiO NPs, meanwhile at both concentrations, the increase resulted after week 2 up to the third week, with respect to unexposed SCs (**Figures 7B**, *p < 0.05 and **p < 0.001 vs. 0 NiO NPs).

### MAPK kinase signaling pathway activation

**Figure 8:**
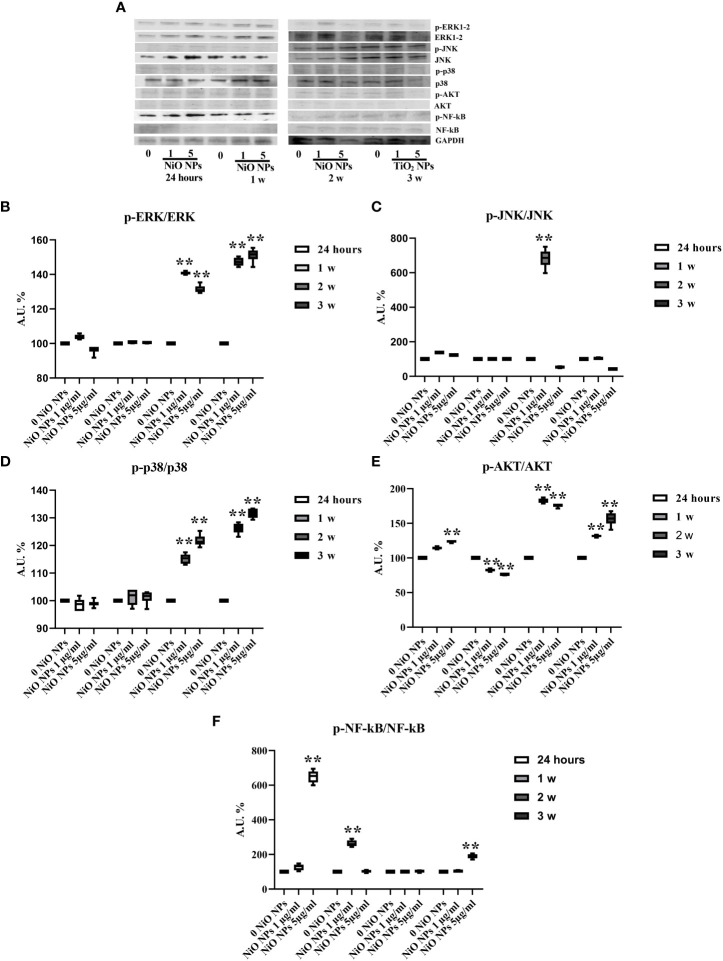
MAPK Kinase Signaling Pathway analysis in WB. **(A)** Immunoblots of phosphoERK1-2/ERK1-2, phosphoJNK/JNK, phosphop38/p38, phosphoAKT/AKT, posphoNF-kB p65/NF-kB, and GAPDH in SCs 24 h and 1, 2, and 3 weeks of incubation with NiO NPs 1 and 5 μg/ml. **(B)** Densitometric analysis of the protein bands of phosphoERK1-2/ERK1-2, **(C)** phosphoJNK/JNK, **(D)** phosphop38/p38, **(E)** phosphoAKT/AKT, and **(F)** phosphoNF-kB p65/NF-kB in SCs 24 h and 1, 2, and 3 weeks of incubation with NiO NPs 1 and 5 μg/ml. Data represent the mean ± SEM (**p < 0.001 vs. 0 NiO NPs of three independent experiments, each performed in triplicate).

We performed Western blotting analysis to investigate the involvement of different MAPK family members (ERK1/2, JNK, p38, AKT ) and NF-kB signaling pathway after NiO NPs exposure (**Figure 8A**). The phosphorylation ratio of ERK1/2 showed a significant increase at both concentrations, from the second up to third week (**Figure 8B**, **p < 0.001 vs. 0 NiO NPs).

The phosphorylation ratio of JNK increased at 24 h in both concentrations, with a significant increase at the second week only at the 1 μg/ml NiO NPs dose and a significant reduction at third week at the 5 μg/ml NiO NPs dose (**Figure 8C**, **p < 0.001 vs. 0 NiO NPs).

The phosphorylation ratio of p38 showed a significant increase from second up to third week, at both NiO NPs doses (**Figure 8D**, **p < 0.001 vs. 0 NiO NPs).

The phosphorylation ratio of AKT showed a significant increase at 24 h, second and third week, with a significant reduction only at first week at both concentrations of treatment (**Figure 8E**, **p < 0.001 vs. 0 NiO NPs).

Finally, the phosphorylation ratio of p-NF-kB showed a significant increase only at first week at dose of 1 μg/ml NiO NPs, meanwhile at dose of 5 μg/ml NiO NPs, the increase resulted after 24 h and at third week of treatment compared to unexposed SCs (**Figures 8F**, **p < 0.001 vs. 0 NiO NPs).

## Discussion

According to World Health Organization estimation, infertility, defined as ‘the inability of a sexually active, non-contracepting couple to achieve spontaneous pregnancy in one year’, affects about 15% of couples of childbearing age in industrialized countries (39). Since a male factor is responsible in about 30% of the cases, and in 20% of cases both male and female factors are involved, about 50% of cases of infertility are related to the male partner (40). Unfortunately, despite progress, the etiology of male infertility is still unknown in 30 to 40% of the cases, thus defined as idiopathic infertility (40, 41). Patients with idiopathic infertility do not have a history compatible with fertility-altering diseases, and their physical, laboratory, genetic, and instrumental examinations are unremarkable; however, their semen frequently shows significant alterations in sperm parameters. Such idiopathic sperm anomalies (such as azoospermia, oligozoospermia, teratozoospermia, and/or asthenospermia) are presumed to be caused by several factors, including reactive oxygen species (ROS), unknown genetic and epigenetic abnormalities, and endocrine disruption due to environmental pollution (42).

As a nanomaterial, NiO NPs are widely used in various fields (43). Humans can be exposed to NiO NPs through environmental and occupational settings. Currently, NiO NPs have been shown to impair the development of reproductive organs, resulting in male infertility.

Notably, in previous studies, it has been demonstrated that NiO NPs cause reproductive toxicity in healthy adult rats, increasing the ratio of epididymis weight to body weight, changing sperm motility parameters in rats, disturbing spermatogenic tubule cells, inducing apoptosis and necrosis (44).

Employing animal models, it was verified that various types of nanoparticles, including NiO NPs, have a negative impact on male germ cells; their damage potential differing in regard to nanoparticle modification, composition, concentration, route of administration, and the species of the animal (2).

During present investigation, an attempt was made to study the influence of acute (24 h) and chronic (from 1 up to 3 weeks) exposure to subtoxic NiO NPs doses of 1 μg/ml and 5 μg/ml on our *“in vitro”* model of SCs.

The NiO NPs doses were chosen according to MTT cytotoxicity assay and, the viability was expressed as a percentage of cells compared to unexposed SCs (NPs-exposed SCs ×100/unexposed SCs).

Due to the evident toxicity of NiO NPs at the 3-week treatment, the sub-toxic concentrations of 1 μg/ml and 5 μg/ml were chosen. Although *in vitro* studies with NPs enable the identification of conceptual models of mechanistic interaction with cells, they do not represent a full realistic model of how NPs will interact with the specific organ of the body *in vivo*. Unfortunately, nowadays no consistent epidemiologic studies exist on the association between reproductive health and the risk of NiO NPs exposure in humans.

In the first analysis, our data demonstrated that the SCs exposed to both subtoxic doses (1 μg/ml and 5 μg/ml) of NiO NPs didn’t show substantial morphological changes.

Oxidative stress is a key contributor to the reproductive toxicity caused by NPs (45). Reactive oxygen species (ROS) are a major factor in inducing 30–80% of infertility issues in men (46), since the increased production of ROS leads to cell apoptosis and impaired spermatogenesis (47).

NiO NPs induced oxidative damage has been demonstrated in different organs of rats (16, 48, 49) and mice (50).

Several studies have investigated the relationship between the depletion of cellular antioxidants in male reproductive organs and infertility (51, 52).

The increase in ROS may induce lipid peroxidation, leading to loss of cell membrane integrity and axonemic damage, reduced sperm viability, and later increased sperm abnormalities (53). It has also been reported that lipid peroxidation alters the germ cell membrane, leading to inhibition of spermatogenesis and cell death, resulting in decreased sperm count (54). Therefore, oxidative stress damage induced by NiO NPs may be the main mechanism of their toxicity, which may be related to the binding of nickel to amino acids, polypeptides and proteins to promote the production of ROS (55).

Our results would agree with these data. In fact, in our model, NiO NPs exposure, at each concentration, induced a marked increase of intracellular ROS at the third week of treatment and DNA damage at all exposure times.

Studies by Kong et al. discovered that NiO NPs reduce the activity of superoxide dismutase (SOD) and catalase (CAT) in rats. Following exposure to NiO NPs, the cell concentration of the antioxidant enzymes SOD and CAT increases in an attempt to counteract the injury caused by ROS. When the antioxidant effect is inadequate to resist the action of ROS, the balance between the production of ROS and the antioxidant system response breaks down, which subsequently leads to reduced levels of antioxidant enzymes, increased ROS content, oxidative stress, and eventually cell death (56).

We measured gene expression of antioxidative enzymes (ROS removal agents) including SOD, HO-1, and GHSPx as downstream molecules of Nrf2/ARE pathway. The SCs exposed to both concentration of NiO NPs showed the upregulation of SOD1 and HO-1, while, the increase of GHSPx was evident only at second week (at 1 μg/ml NiO NPs) and first week (at 5 μg/ml NiO NPs).

We might hypothesize that the Nrf2/ARE pathway activation was enough to cope with ROS production only during the acute exposure to subtoxic doses of NiO NPs; meanwhile, its activation was not able to counteract the oxidative stress generated throughout the chronic exposure at the subtoxic toxic dose.

The effects on SCs exposed to NiO NPs toxicity were evaluated using functional biomarkers of these cells, such as the gene expression and secretion of AMH and inhibin B.

AMH is a dimeric glycoprotein that belongs to the transforming growth factor-β (TGF-β) superfamily, which includes inhibin B, activins, and others (57). It is exclusively secreted by SCs, thus representing a useful markers of testis functionality during the pre‐pubertal period (58). Inhibin B is a heterodimeric glycoprotein, which plays a role in the negative feedback control of FSH secretion in men (59). Inhibin B is a marker used in clinical practice to evaluate the presence and function of SCs during childhood (60). We observed that AMH and inhibin B gene expression and secretion significantly decreased up to the third week at both concentrations of NiO NPs-exposure. This result is an expression of the reduced Sertolian functionality caused by subtoxic doses of NiO NPs chronic exposure on our SCs model.

We then evaluated the activation of apoptosis assessing the caspase-3 protein expression.

Magaye and Zhao discovered that NiO NPs is able to induce genotoxicity by switching on apoptosis-related genes. Apoptosis induced by NiO NPs mainly engages the death receptor-mediated pathway and the mitochondria-mediated pathway (9). Kong et al. found that NiO NPs increased the levels of pro-apoptotic factors, such as caspase-3, caspase-8, caspase-9 and reduced the levels of anti-apoptotic factor Bcl-2 protein. The apoptosis process mediated by caspase-3 can be triggered by p53 activated by NiO NPs (61). Our results showed the activation of caspase-3 during third week of treatment with 5 μg/ml NiO NPs, with increase of both active p19 and p17 compared to the inactive p35 fragments, expression of a more prominent apoptotic process.

Regarding inflammation, administration of NiO NPs in mice alters the balance between pro-inflammatory and anti-inflammatory response (62), during which monocyte-differentiated macrophages produce pro-inflammatory cytokines such as IL-1β, IL-6, and TNF-α (63). After intratracheal instillation of the same concentration of NiO NPs, various cytokines were found to work as proliferation and/or survival drivers, and TNF-α and IL-6 were also significantly increased in all experimental groups compared with the control group. Accordingly, it is possible to suppose that up-regulation of pro-inflammatory effects of IL-1β, IL-6, and TNF-α may deepen the inflammatory reaction and microstructure damage of testicular tissues.

We were able to observe, at both subtoxic doses of NiO NPs a clear pro-inflammatory stress with the steady increase in the gene expression of TNF-α and IL-6.

The MAPK signal transduction pathway, also known as the mitogen-activated protein kinase pathway, includes three parallel pathways, namely the ERK pathway, the JNK/SAPKK pathway and the P38MARK pathway (64). Being some of the most widely used transcription factors in cells, they are implicated in many significant cellular activity processes, including proliferation, differentiation, and apoptosis (65).

Magaye et al. reported that NiO NPs at concentrations of 0, 2.5, 5, 7.5, and 10 µg/cm2 significantly up-regulated the protein expression of phosphorylated ERK1/2 (p-ERK1/2), phosphorylated JNK (p-JNK) and phosphorylated P38 (p-P38) (6, 7).

SCs treated with both concentrations of NiO NPs markedly increased the phosphorylation ratio of p-ERK1/2, p-38 and p-AKT from the second up to the third week of treatment, as a response to a state of inflammation and apoptosis.

This study points out the importance of deepening the effects of the chronic exposure to subtoxic doses of NiO NPs on *“in vitro”* model of SCs, underlining that to identify damages in the Sertolian pre-puberal phase is crucial to predict future irreversible alterations of spermatogenesis in adulthood.

The *limitation* of this study is represented by the difficulty of to isolate (if not impossible) adult SC because of very tight intercellular junctions.

Disruption of such junctions, during testis digestion, severely damages SC viability. In fact, adult Sertolian cell lines are commonly used (66), that are very far from simulating physiological characteristics of SC when investigated as primary cultures (67).

Obvioulsly, pre-pubertal human SC are quite difficult to find and harvest and, above all, raise unsolved ethical problems (in fact, many Countries, including ours, prohibits retrieval of reproductive organs from cadavers).

The use of SCs does not impact on results.

## Conclusions

The present study has concluded that the chronic exposure to subtoxic doses of NiO NPs induces adverse effects on SCs functionality and viability. Our *in vitro* pilot study could help to adopt future containment strategies and active surveillance programs, as preventive measures before irreversible damage to SCs may occur and consequently affects spermatogenesis.

## Data availability statement

The original contributions presented in the study are included in the article/**Supplementary Materials**. Further inquiries can be directed to the corresponding author.

## Ethics statement

Animal studies were conducted in agreement with the guidelines adopted by the Italian Approved Animal Welfare Assurance (A-3143-01) and the European Communities Council Directive of November 24, 1986 (86/609/EEC).

## Author contributions

IA designed and drafted the manuscript. The experimental procedures and data analysis were performed by AM, CB, CL, MA, DB, MC, EE, FG and TB. SG, AG, and GM gave experimental guidance. FM and GL supervised and revised the manuscript. All authors contributed to the article and approved the submitted version.

## Supplementary material

Supplementary Figure 1 Uptake of NiO NPs by inductively coupled plasma-optical emission spectrometry (ICP-OES). Percentage of internalized nanoparticles (% uptake) and amount of metal adsorbed per cell number (expressed as ng/105) in SCs 5 h of incubation with NiO NPs 1 and 5μg/ml. Data represented as mean ± SEM of three independent experiments, each are performed in triplicate.

Supplementary Figure 2 NiO NPs Cytotoxicity Evaluation by MTT test. **(A)** Evaluation of NiO NPs toxicity in SCs at 24 (grey line) and 48 hours (black line) of incubation with NiO NPs 2.5, 5, 15, 30, 45, 60 and 120 µg/ml. **(B)** Evaluation of NiO NPs toxicity in SCs at 24 hours, 1, 2 and 3 weeks of incubation with NiO NPs 1 (grey line) and 5 µg g/ml (black line). Data represented as mean ± SEM. (*p<0.05 and **p<0.001 *vs* unexposed SCs of three independent experiments, each performed in triplicate).
