# Corrigendum: Nickel oxide nanoparticles exposure as a risk factor for male infertility: “*In vitro*” effects on porcine pre-pubertal Sertoli cells

**DOI:** 10.3389/fendo.2025.1625122

**Published:** 2025-06-19

**Authors:** Iva Arato, Stefano Giovagnoli, Alessandro Di Michele, Catia Bellucci, Cinzia Lilli, Maria Chiara Aglietti, Desirée Bartolini, Angela Gambelunghe, Giacomo Muzi, Mario Calvitti, Elena Eugeni, Francesco Gaggia, Tiziano Baroni, Francesca Mancuso, Giovanni Luca

**Affiliations:** ^1^ Department of Medicine and Surgery, University of Perugia, Perugia, Italy; ^2^ Department of Pharmaceutical Sciences, University of Perugia, Perugia, Italy; ^3^ Department of Physics and Geology, University of Perugia, Perugia, Italy; ^4^ Internal Medicine Endocrine and Metabolic Sciences Unit, Santa Maria della Misericordia Hospital of Perugia, Perugia, Italy; ^5^ International Biotechnological Center for Endocrine, Metabolic and Embryo-Reproductive Translational Research (CIRTEMER), Department of Medicine and Surgery, University of Perugia, Perugia, Italy; ^6^ Division of Medical Andrology and Endocrinology of Reproduction, Saint Mary Hospital, Terni, Italy

**Keywords:** Sertoli cells, nickel oxide nanoparticles, ROS, comet, MAPK pathways

In the published article, there was an error in **Figure 6** panel A as published. Duplicate image for four loading controls that were used by our group one year before in Figure 5A of Mancuso et al., 2022, where the conditions are different (TiO2-NPs). The corrected **Figure 6** panel A and its caption “Caspase-3 Evaluation by WB analysis. (A) Immunoblots of caspase-3 p35, p19, and p17 in SCs at 24h and 1, 2, and 3 weeks of incubation with NiO-NPs at 1 and 5mg/ml” appear below.

**Figure 6:**
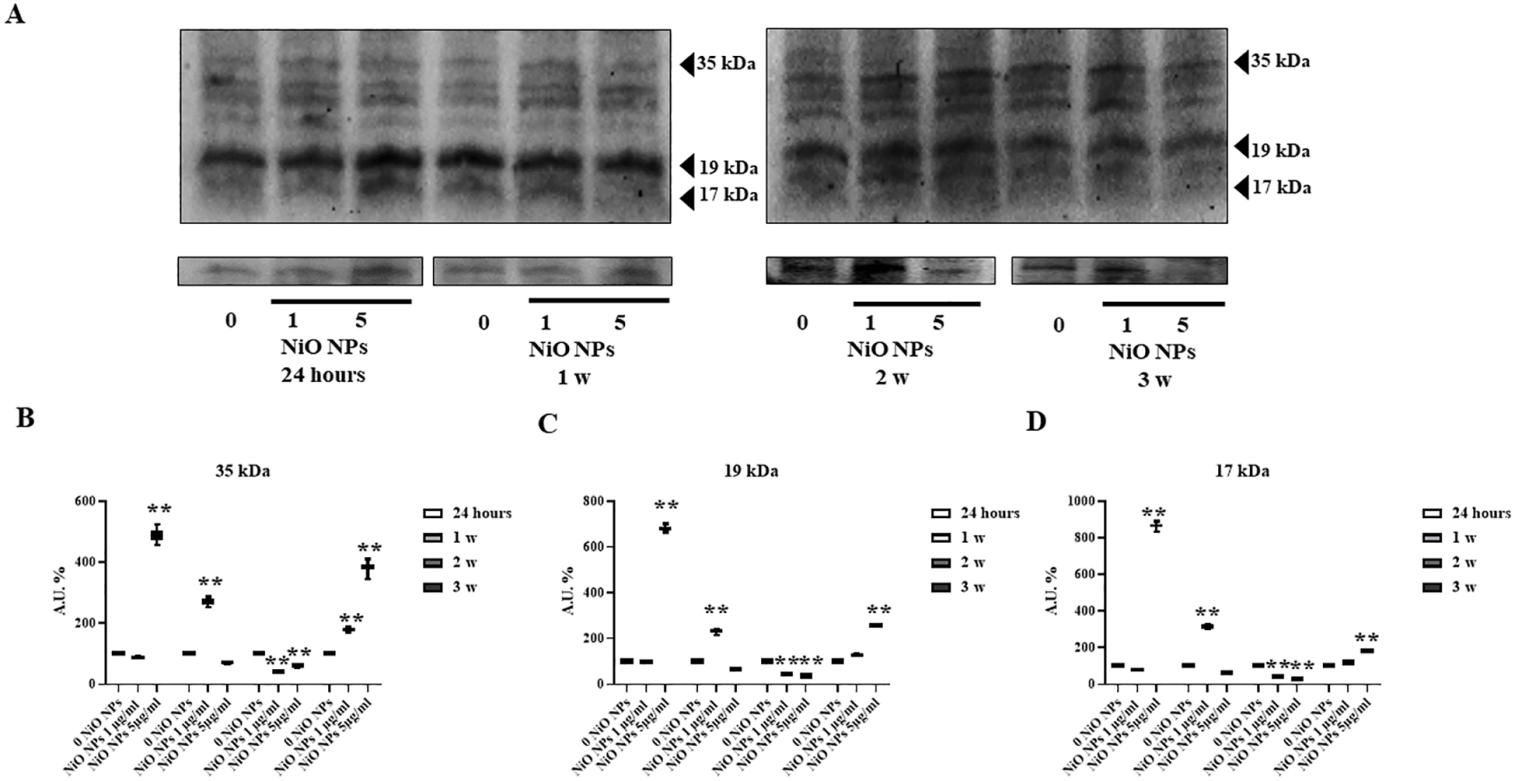
Caspase-3 Evaluation by WB analysis. **(A)** Immunoblots of caspase-3 p35, p19, and p17 in SCs at 24h and 1, 2, and 3 weeks of incubation with NiO-NPs at 1 and 5mg/ml. Densitometric analysis of the protein bands of caspase-3 p35 **(B)**, p19 **(C)**, and p17 **(D)** in SCs at 24 h and 1, 2, and 3 weeks of incubation with NiO NPs 1 and 5 μg/ml. Data represent the mean ± SEM (**p < 0.001 vs. 0 NiO NPs of three independent experiments, each performed in triplicate).

The authors apologize for this error and state that this does not change the scientific conclusions of the article in any way. The original article has been updated.

