# Supplementary figures and images for: Nickel oxide nanoparticles exposure as a risk factor for male infertility: “In vitro” effects on porcine pre-pubertal Sertoli cells
Source: Front Endocrinol (Lausanne). 2023 Mar 30;14:1063916. doi: 10.3389/fendo.2023.1063916 (PMC10098343; doi:10.3389/fendo.2023.1063916)

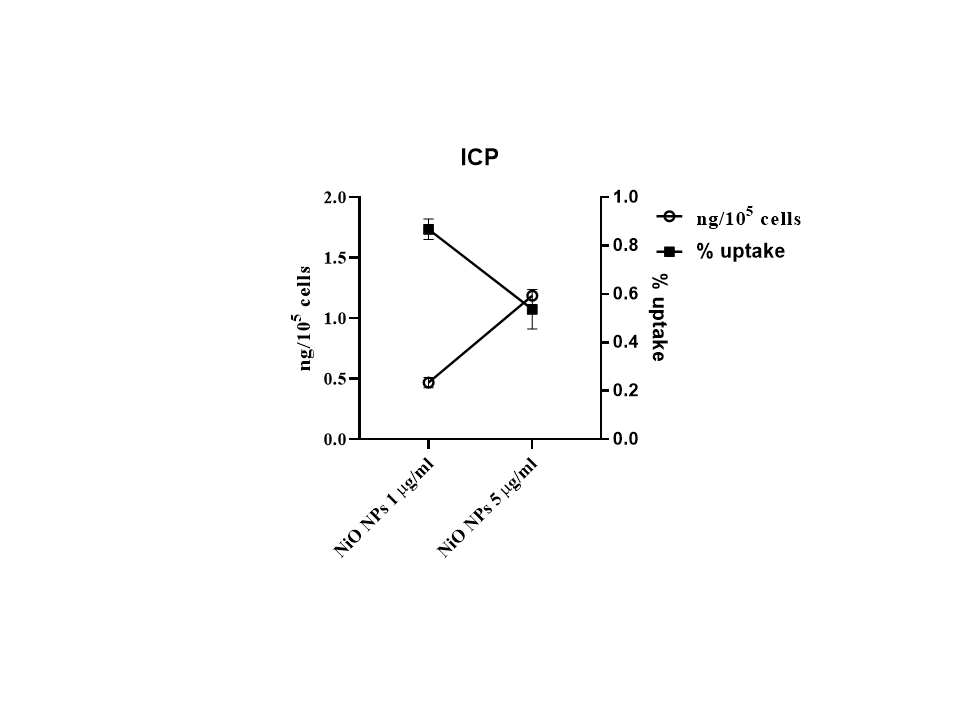

Supplement: Supplementary Figure 1 — Uptake of NiO NPs by inductively coupled plasma-optical emission spectrometry (ICP-OES). Percentage of internalized nanoparticles (% uptake) and amount of metal adsorbed per cell number (expressed as ng/105) in SCs 5 h of incubation with NiO NPs 1 and 5μg/ml. Data represented as mean ± SEM of three independent experiments, each are performed in triplicate. [file Image_1.tif]

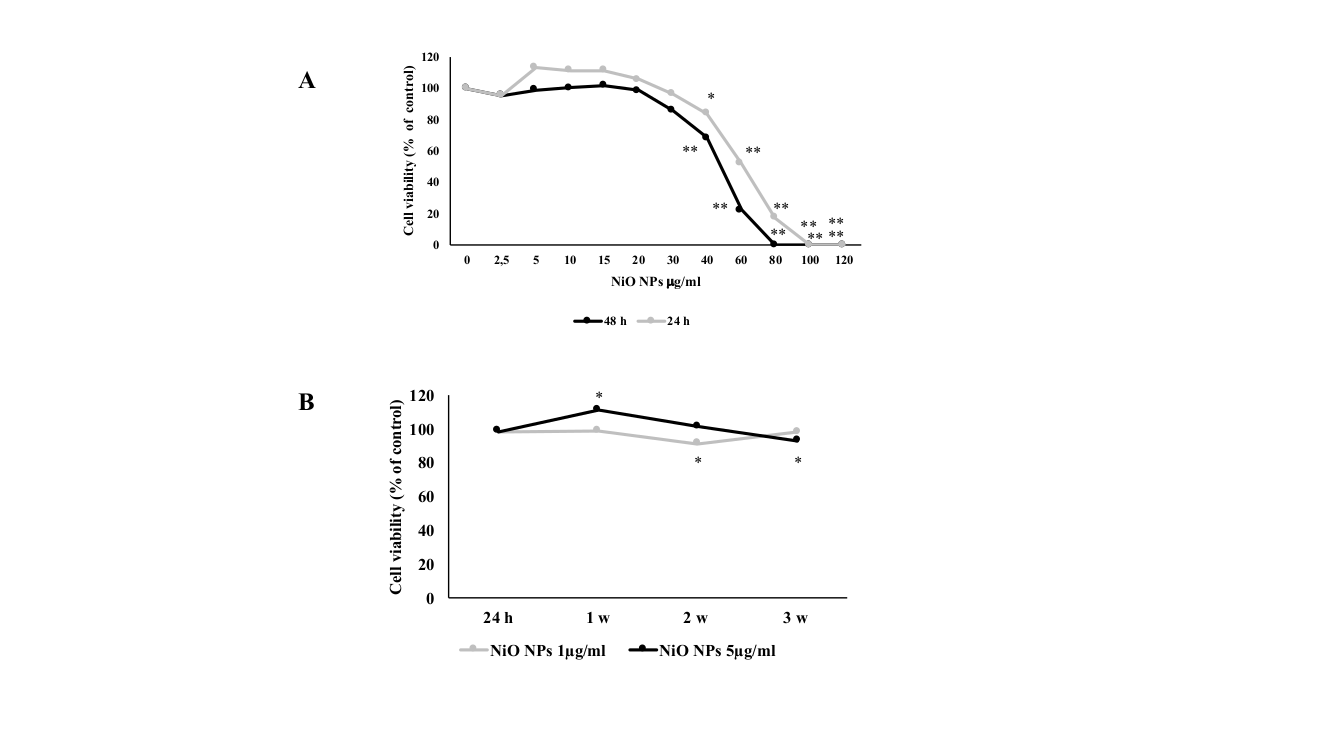

Supplement: Supplementary Figure 2 — NiO NPs Cytotoxicity Evaluation by MTT test. (A) Evaluation of NiO NPs toxicity in SCs at 24 (grey line) and 48 hours (black line) of incubation with NiO NPs 2.5, 5, 15, 30, 45, 60 and 120 µg/ml. (B) Evaluation of NiO NPs toxicity in SCs at 24 hours, 1, 2 and 3 weeks of incubation with NiO NPs 1 (grey line) and 5 µg g/ml (black line). Data represented as mean ± SEM. (*p<0.05 and **p<0.001 vs unexposed SCs of three independent experiments, each performed in triplicate). [file Image_2.tif]
